# Transplantation of human-induced pluripotent stem cells carried by self-assembling peptide nanofiber hydrogel improves bone regeneration in rat calvarial bone defects

**DOI:** 10.1038/bdjopen.2015.7

**Published:** 2016-01-29

**Authors:** Kamichika Hayashi, Hiromi Ochiai-Shino, Takeaki Shiga, Shoko Onodera, Akiko Saito, Takahiko Shibahara, Toshifumi Azuma

**Affiliations:** 1 Department of Oral and Maxillofacial Surgery, Tokyo Dental College, Tokyo, Japan; 2 Department of Biochemistry, Tokyo Dental College, Tokyo, Japan

## Abstract

**Objectives/Aims:**

The requisite conditions for successful bone tissue engineering are efficient stem cell differentiation into osteogenic cells and a suitable scaffold. In this study, we investigated *in vivo* bone regeneration from transplanted induced pluripotent stem cells (iPSCs).

**Materials and Methods:**

Two critical-sized calvarial bone defects were created in 36 rats. The surgical sites were randomly assigned to one of three treatments to test the healing effectiveness of the scaffold alone, scaffold with iPSCs or a salt solution as a control. The effectiveness of the treatments was evaluated after 2 or 4 weeks using radiographic and histological analyses of bone regeneration in the six groups.

**Results:**

Micro-computed tomography (CT) analysis of the bone defects found minimal bone regeneration with the salt solution and nanofiber scaffold and increased bone regeneration in defects repaired with iPSCs delivered in the nanofiber scaffold.

**Conclusion:**

Transplanted iPSCs encapsulated in a nanofiber scaffold can regenerate bone in critical-sized defects.

## Introduction

Bone grafting is often performed to fill bone defects and promote bone regeneration.^1^ Fresh autologous grafts remain the ‘gold standard’ for stimulating bone repair and regeneration,^2^ but their availability may be limited and the procedure used to collect this material is associated with complications. This approach is not suitable for large defects and it has several risks, including donor site morbidity, graft or flap failure, rejection or infection.^3^ In general, artificial bones made of various materials^4,5^ are used because people still have difficulty in accepting the use of allografts.^6^

Artificial bone has many advantages and several limitations because grafted artificial bone can be integrated into the host bone without rejection to obtain sufficient strength. The artificial bone should also act as a carrier or scaffold for cell growth to prevent scar formation and promote bone regeneration.^7^ Thus, it is important to overcome limitations such as limited availability and the invasiveness of the procedures, which are often associated with many problems.^5,8,9^

Bone tissue engineering^9^ is a relatively new method for repairing damaged bones.^5,9^ This concept involves the regeneration of tissues using stem cells, scaffolds and growth factors, with stem cells playing a leading role in tissue regeneration. Recent studies have confirmed that biological factors such as growth factors and cells also have crucial roles in tissue regeneration. Several studies^5,10^ have indicated that a combination of various signalling molecules (growth factors and cytokines) is better for optimising bone regeneration, and different mixtures of two or more factors have been investigated in bone regeneration analyses.^11,12^

Bone marrow mesenchymal stem cells (MSCs)^13^ have great potential for bone regeneration, and clinical applications of MSCs are being developed.^14^ These studies showed that the paracrine effects of growth factors and cytokines secreted by transplanted MSCs may promote tissue repair and regeneration and, thus, transdifferentiation of the transplanted cells.^10,15,16^ In recent years, many studies have shown that induced pluripotent stem cells (iPSCs) are remarkable materials in regenerative medicine.^17–22^ Previously, we reported that human iPSCs (hiPSCs) can differentiate into osteoblasts and osteocytes.^23^

In bone tissue engineering, cells and growth factors are combined with a porous biodegradable scaffold to repair and regenerate tissue. The scaffold acts as a temporary matrix while the cells secrete the extracellular matrix that is required for tissue regeneration. Scaffolds can be used to induce the formation of desired tissue following the growth of cells from surrounding areas and as carriers for seeded autogenous cells that are cultured in bioreactors and subsequently reimplanted into the host.^2,4,5,7^ As a material that promotes the functioning of cells as an extracellular matrix, we considered the use of self-assembling peptide nanofiber hydrogel, a synthetic peptide consisting of a 16 amino-acid sequence.^24^

The requisite conditions for successful bone tissue engineering are efficient stem cell differentiation into osteogenic cells and a suitable scaffold. In this study, we investigated the *in vivo* bone regeneration of transplanted iPSCs.

## Materials and methods

### Cell culture and embryoid body formation, *in vitro* differentiation and cell sorting

**Figure 1:**
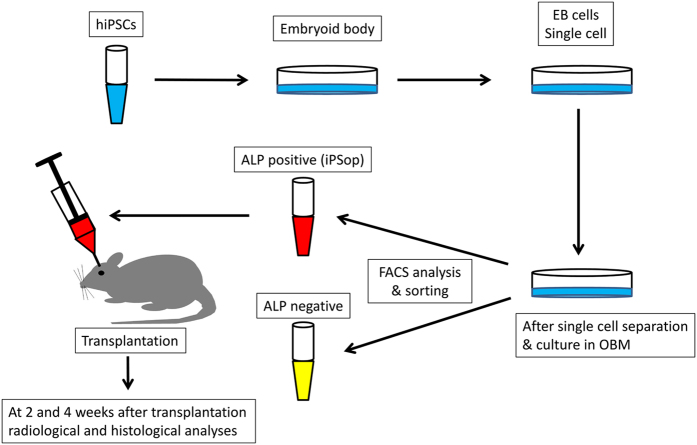
Outline of the experimental protocol. Embryoid bodies (EBs) were cultured on low-attachment Petri dishes for 6 days and dissociated in 0.5 mg/ml collagenase type IV and 0.05% trypsin-EDTA. The trypsinised EBs were then cultured in osteoblast differentiation medium (OBM) on cell culture dishes. The next day, various cytokines were added to the dishes (day 0) and the OBM containing cytokines was changed every 3 days. After 14 days, the cells were analysed and isolated by fluorescence-activated cell sorting. At 2 and 4 weeks after transplantation, the animals were radiographically and histologically analysed. ALP, alkaline phosphatase.

Embryoid bodies were cultured on low-attachment Petri dishes for 6 days and were then dissociated in 0.5 mg/ml collagenase type IV (Wako Pure Chemical Industries, Osaka, Japan) and 0.05% trypsin-EDTA (Invitrogen, Carlsbad, CA, USA). A schematic representation of the protocol for the differentiation of hiPSCs (line 201B7; Riken Cell Bank, Tsukuba, Japan) into osteoblast-like cells is shown in Figure 1. The next day, various cytokines were added to the dishes (day 0) and the osteoblast differentiation medium containing cytokines was changed every 3 days.^12^ After 2 weeks, the hiPSC-derived embryoid bodies that had differentiated during culture in osteoblast differentiation medium were dissociated with 0.05% trypsin-EDTA for 10 min at 37 °C. The trypsinised hiPSCs were stained with phycoerythrin conjugated to anti-human alkaline phosphatase antibody (R&D Systems, Minneapolis, MN, USA) for 45 min on ice. After staining, the cells were washed three times with phosphate-buffered saline, suspended in phosphate-buffered saline containing 0.5% fetal bovine serum, passed through a 40-μm-mesh filter and maintained at 4 °C until flow cytometric analysis. Dead cells were excluded from the flow cytometric analysis based on propidium iodide staining (2 μg/ml) and forward scatter. Flow cytometric analysis and cell sorting were performed using a FACSAria system (Becton-Dickinson, San Jose, CA, USA). The cells obtained from cell sorting were used as hiPSC-derived osteoprogenitors (iPSop cells; Figure 1).

### Three-dimensional hydrogel

To facilitate the self-assembly of a peptide nanofiber scaffold-derived three-dimensional (3D) culture, we used PuraMatrix (BD Biosciences, Cambridge, MA, USA) to encapsulate the cells.^24,25^ Five microlitres of freshly dissociated cells suspended in serum-free media was mixed with 45 μl of the peptide in the wells of a 24-well plate. Gelation was initiated after the peptide solution was mixed with the cell suspension, thereby resulting in cell encapsulation inside the nanofiber hydrogel. Approximately, 500 μl of serum-free basal osteoblast differentiation medium was added to neutralise the acidic hydrogel environment.

### Rat calvarial bone defect model

**Figure 2:**
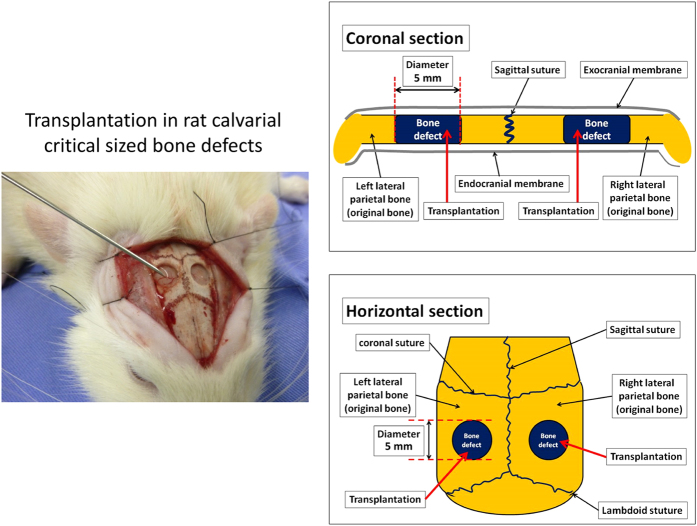
Transplantation protocol for rat calvarial critical-sized bone defects. A linear sagittal incision was made along the top of the skull, followed by full thickness retraction of the skin and periosteum to expose the calvarium. Critical-sized bone defects (diameter=5 mm) were created in the dorsal area. After transplantation, the periosteal flap was closed by suturing.

All of the procedures performed with live animals conformed to the ethical guidelines established by the Japanese Council on Animal Care and were approved by the animal care committee of the Tokyo Dental College (Permit Number: 11–324, 12–274). Fourteen-week-old male Sprague-Dawley rats (*n*=36) were obtained from Sankyo Laboratory (Tokyo, Japan). After anaesthesia induction with 4% sevoflurane (Maruishi Pharmaceutical, Osaka, Japan) inhalation, the rats were further anaesthetised by intraperitoneal injection with sodium pentobarbital (30 mg/kg body weight Somnopentyl; Kyoritsu Seiyaku, Tokyo, Japan). A linear sagittal incision was then made along the top of the skull, followed by full thickness retraction of the skin and periosteum to expose the calvarium. Critical-sized bone defects (diameter=5 mm) were created in the dorsal area. After transplantation, the periosteal flap was closed by suturing (Figure 2).

Two critical-sized calvarial bone defects were created in 36 rats. The surgical sites were randomly assigned to one of three treatments to test the healing effectiveness of the nanofiber scaffold alone (nanofiber), nanofiber scaffold with iPSCs (nanofiber+iPSCs) or a physiological salt solution (saline) as a control. The effectiveness of the treatments was evaluated after 2 or 4 weeks using radiographic and histological analyses of bone regeneration in the six groups.

**Figure 3:**
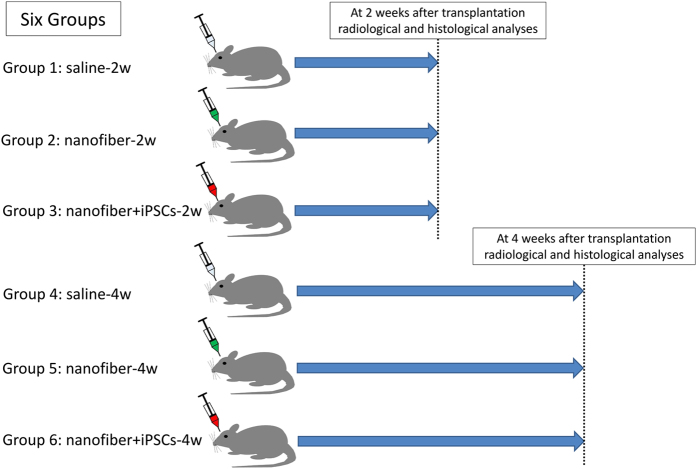
Experimental groups. The rats were randomly assigned to six groups. The saline-2w, nanofiber-2w and nanofiber+iPSCs-2w groups were radiographically and histologically analysed at 2 weeks after transplantation, whereas the saline-4w, nanofiber-4w and nanofiber+iPSCs-4w groups were radiographically and histologically analysed at 4 weeks after transplantation.

The experimental groups were as follows: saline-2w and saline-4w groups, in which the two defects were filled with physiological salt solution (10 μl) alone; nanofiber-2w and nanofiber-4w groups, in which the two defects were filled with self-assembling peptide nanofiber hydrogel (10 μl) alone; and nanofiber+iPSCs-2w and nanofiber+iPSCs-4w groups, in which the two defects were filled with iPSop cells (1×10^5^ cells) encapsulated in self-assembling peptide nanofiber hydrogel (10 μl). The saline-2w, nanofiber-2w and nanofiber+iPSCs-2w groups were radiographically and histologically analysed at 2 weeks after transplantation, whereas the saline-4w, nanofiber-4w and nanofiber+iPSCs-4w groups were radiographically and histologically analysed at 4 weeks after transplantation (Figure 3). The rats were immunosuppressed using FK-506 (Astellas Pharma, Tokyo, Japan), which was administered after surgery at a dose of 2 mg/kg per day every day.^26^

### Radiographic analyses

**Figure 4:**
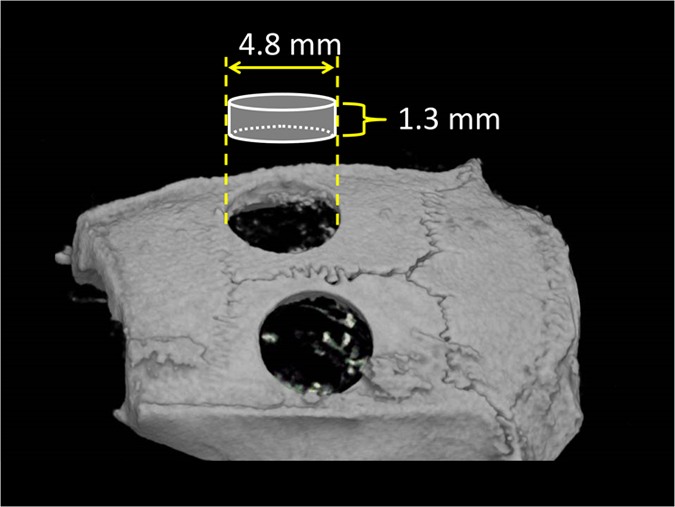
Area visualised by TRI/3D-BON. Micro-CT parameters were as follows: X-ray source, 85 kV/140 μA; rotation, 360°; exposure time, 17 s; voxel size, 50×50×50 μm (R-mCT; Rigaku). CT images were compiled and 3D images were rendered using TRI/3D-BON (Ratoc System Engineering). The software was used to obtain a 3D reconstruction of the sets of scans. From the overall 3D data set, a region of interest with a diameter of 4.8 mm and height of 1.3 mm was selected for analysis, which included the entire thickness of the calvarial bone.

At 2 and 4 weeks after surgery, CT images were compiled and 3D images were rendered using TRI/3D-BON (Ratoc System Engineering, Tokyo, Japan). This software was used to obtain a 3D reconstruction from the sets of scans. Micro-CT parameters were as follows: X-ray source, 85 kV/140 μA; rotation, 360°; exposure time, 17 s; voxel size, 50×50×50 μm (R-mCT; Rigaku, Tokyo, Japan). From the overall 3D data set, a cylindrical region of interest with a diameter of 4.8 mm and height of 1.3 mm was selected for analysis, which included the entire thickness of the calvarial bone (Figure 4). 3D images of the new bone formation were displayed and the bone volume (μm^3^) was measured.

### Histological analyses

At 2 and 4 weeks after transplantation, the animals were killed by deep anaesthesia using sodium pentobarbital. The skin was dissected and the defect sites were removed, along with the surrounding bone and soft tissues. Coronal sections (thickness=5 μm) through the centre of each circular defect were prepared and routine histological haematoxylin and eosin (H&E) staining and Villanueva-Goldner (V-G) staining^27^ were performed. For H&E staining, 5-μm-thick paraffin sections were obtained, placed on microscope slides and deparaffinised with xylene. After removing the paraffin, we rehydrated the sections with an ethanol/phosphate-buffered saline series (100% ethanol, 95% ethanol and 70% ethanol) and finally immersed them in phosphate-buffered saline. Sections stained with H&E and V-G were analysed by light microscopy.

### Statistical analyses

The data are expressed as the mean±s.d. and were analysed with analysis of variance and Bonferroni tests. All of the data represent at least three independent experiments. *P*<0.05 was considered to be statistically significant. Statistical analyses were performed using the SPSS software package (version 15 for Windows; SPSS, Chicago, IL, USA).

## Results

### Micro-CT observations

**Figure 5:**
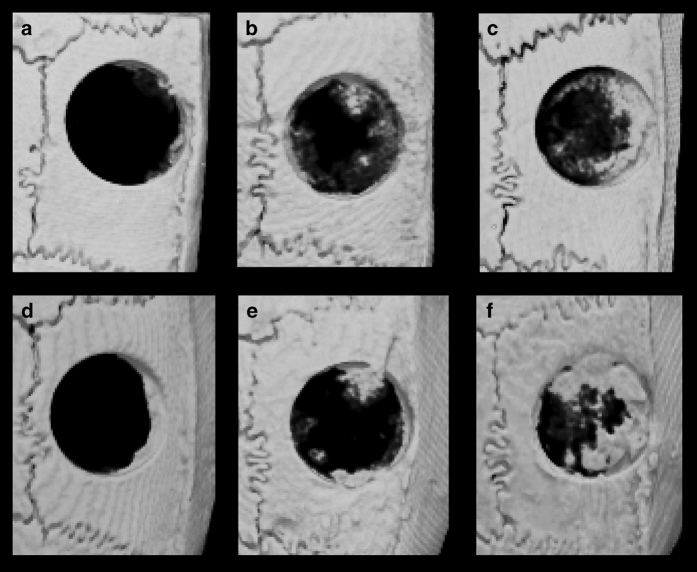
Micro-CT images of the calvaria after transplantation. (**a**) Saline-2w group. (**b**) Nanofiber-2w group. (**c**) Nanofiber+iPSCs-2w group. (**d**) Saline-4w group. (**e**) Nanofiber-4w group. (**f**) Nanofiber+iPSCs-4w group.

Micro-CT images of the calvaria at 2 and 4 weeks after transplantation are shown in Figure 5. Re-ossification developed via growth extension from the bony rims at the lateral edges of the bone defects. Newly generated bone was observed as early as 2 weeks after surgery in the nanofiber-2w and nanofiber+iPSCs-2w groups. Minimal new bone was observed in the saline-2w group. Compared with the saline-4w group, the bone volume was significantly higher in the nanofiber+iPSCs-4w group at 4 weeks after transplantation. The micro-CT images showed that bone formation occurred uniformly from the bony rims at the lateral edges of the bone defects in the nanofiber+iPSCs-2w and nanofiber+iPSCs-4w groups. In the nanofiber+iPSCs-2w and nanofiber+iPSCs-4w groups, the images showed that a hard tissue layer had formed on the bone defect, as well as between the periosteum and the lateral parietal bone (Figure 5).

**Figure 6:**
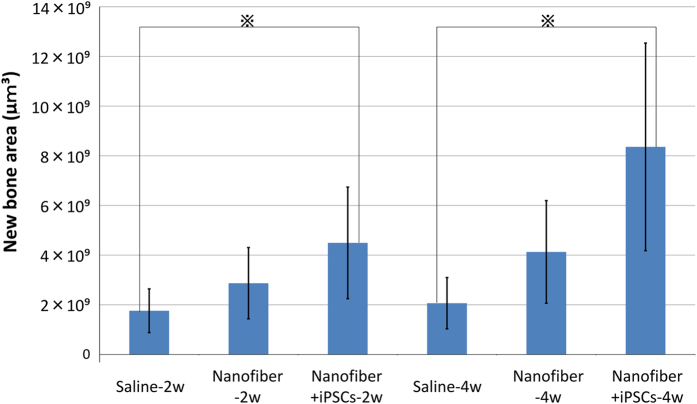
Micro-CT analysis of bone regeneration. Comparison of new bone volume (μm^3^) in a region of interest of six groups. The data are expressed as the mean±s.d. and were analysed with analysis of variance and Bonferroni testing. All of the data represent at least three independent experiments. *P*<0.05 was considered to be statistically significant. ※*P*<0.05.

The nanofiber+iPSCs-2w group had significantly better regeneration than the saline-2w group (*P*<0.05). The nanofiber+iPSCs-4w group had significantly better regeneration than the saline-4w group (*P*<0.05; Figure 6).

### Histological findings

#### H&E staining

**Figure 7:**
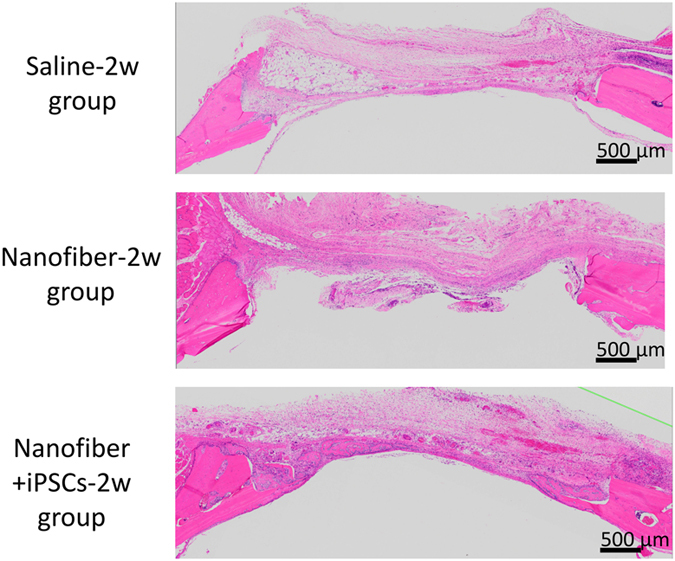
H&E-stained histological images at 2 weeks after transplantation. Top: saline-2w group. Middle: nanofiber-2w group. Lower: nanofiber+iPSCs-2w group.

**Figure 8:**
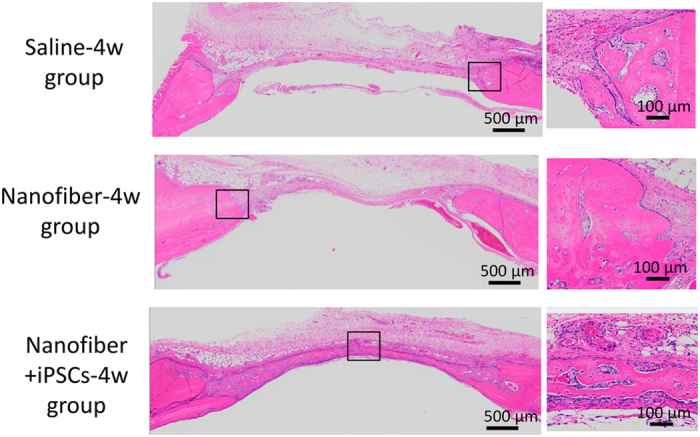
H&E-stained histological images at 4 weeks after transplantation. Top: saline-4w group. Middle: nanofiber-4w group. Lower: nanofiber+iPSCs-4w group. Magnified images of the new bone area are shown on the right for the saline-4w, nanofiber-4w and nanofiber+iPSCs-4w groups.

Images of the calvarial histology in coronal H&E-stained sections are shown in Figures 7 and 8. These photomicrographs of the defects at 2 weeks and 4 weeks were obtained at low magnification in the six groups. In the saline-2w group, granulation tissue and congested vessels were found in the defect area during the second week post surgery. In the fourth week, the defect area exhibited healing with fibrous connective tissue. In both weeks, there was almost no regeneration of the bone in the saline-4w and saline-2w groups (Figures 7 and 8). In the nanofiber-2w and nanofiber-4w groups, the defect area exhibited granulation tissue and the matrix appeared in the second week. No obvious nanofiber hydrogel was visible in the area observed. In the fourth week, fibrous tissue was visible in the defect area, including osteoblasts and some new bone tissue (Figures 7 and 8). In the nanofiber+iPSCs-2w and nanofiber+iPSCs-4w groups, bone tissue was observed in the centre of the defect in the second week, as well as in the marginal rim of the defective area. Medullary cavities were present in the regenerated bone tissues, which indicated mature bone tissue. In the nanofiber+iPSCs-4w group, there were numerous capillaries around the regenerated bone (Figures 7 and 8).

#### V-G staining

**Figure 9:**
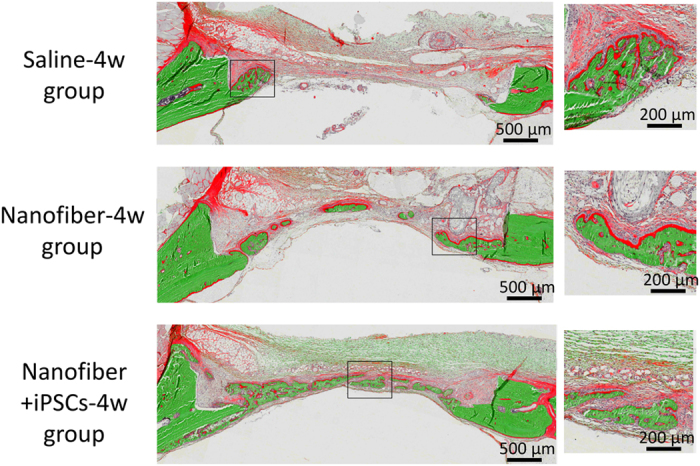
V-G-stained histological images at 4 weeks after transplantation. Top: saline-4w group. Middle: nanofiber-4w group. Lower: nanofiber+iPSCs-4w group. Magnified images of the new bone area are shown on the right for the saline-4w, nanofiber-4w and nanofiber+iPSCs-4w groups.

V-G staining provides uniform and reproducible results with mineralised or undecalcified bone. Thus, this staining method is suitable for evaluating new bone formation. Mineralised bone tissues are stained green and nonmineralised osteoid tissues are stained red by V-G stain. In the saline-4w group, some newly formed bone tissue was observed, which was stained green. In contrast, red staining was observed on the top border of the newly formed bone. In the nanofiber-4w group, newly formed bone was evident around the marginal region of the defective area. These newly formed bone tissues exhibited many medullar-like cavities with marginal red staining. This marginal red staining was more evident on the surface regions of newly formed bones, which indicated that the nanofiber-4w group possessed many osteoid tissues. The newly formed bone was observed around the margin and along the intracranial periosteum, but also in the centre of the bone defects, including around the top portion of the defect area in the nanofiber+iPSCs-4w group. Notably, the newly formed bone exhibited little red staining, indicating that the newly formed bone contained low amounts of osteoid. In the nanofiber+iPSCs-4w group, the newly regenerated bone was also accompanied by less osteoid (Figure 9).

## Discussion

This study is one of the first investigations of the ability of transplanted iPSCs delivered in a nanofiber scaffold to regenerate bone in critical-sized defects. The rat calvarial bone defect model used in our study was obtained by removing hard tissue only from outside the calvaria and, thus, the intracranial periosteum was preserved.^28^ It is well known that the peripheral margins of defects and the intracranial periosteum have the capacity to induce bone formation. Therefore, newly formed bones are mainly observed around the margins of the bone defects and the intracranial periosteum and large bone defects do not heal themselves.^5,7,28^ To overcome this problem, many studies have focused on using various types of biomaterials and cells to facilitate bone regeneration in the centre of bone defects.^29,30^ Artificial bones made of hydroxyapatite or calcium phosphate have often been used to fill bone defects,^4^ but these materials do not allow cells to penetrate adequately or to form stable associations with adjacent bone tissue.^24,25^

Therefore, cells have been applied with biomaterials to fill bone defects in several studies.^1,5^ MSCs have been tested in many studies using calvarial bone defect models, with positive results.^30,31^ However, MSCs are associated with many problems, including high cost and safety concerns related to the invasive procedures used to collect the cells.^31–33^ In contrast, iPSCs have a high potential for proliferating and differentiating into the required tissues and cells, and they are widely expected to be ideal stem cells for tissue-engineering applications.^22,34–37^ Indeed, the clinical application of iPSCs may be beneficial in terms of cost, safety and cell-handling issues, as well as eliminating invasive collection procedures.^34–37^ One of the most important requirements to support the use of iPSCs in tissue engineering to correct bone defects is the establishment of methods for generating osteogenic cells from hiPSCs.

Previously, we reported an efficient method for generating osteoprogenitors from hiPSCs *in vitro*.^23^ Thus, in the present study, we examined the effectiveness of osteoblast transplantation with PuraMatrix, which is a self-assembling synthetic peptide scaffold. Self-assembling peptide nanofiber hydrogel has been used in calvarial bone defect models, where it exhibited acceptable regenerative properties and usefulness.^5,24^ Self-assembling peptide nanofiber hydrogel facilitates the invasion of vascular endothelia cells from adjacent tissues and the formation of new bone tissues.^38,39^ One of the main problems with bone tissue-engineering approaches is the availability of an adequate blood supply.^5,7^ Thus, MSCs cannot survive for a long time in bone defects and they usually disappear within a few weeks of transplantation.^13,14^ We hypothesised that transplanted cells might survive better in self-assembling peptide nanofiber hydrogel than in other conditions.

Another issue related to regeneration in tissue engineering is the importance of the paracrine mechanisms triggered by growth factors and cytokines secreted by transplanted stem cells.^10,15,16,31^ MSCs secrete growth factors and cytokines that support the regeneration of new bone tissues.^31^ We found that iPSop cells encapsulated in self-assembling peptide nanofiber hydrogel facilitated better regeneration and vascularisation than self-assembling peptide nanofiber hydrogel alone. This finding suggests that the presence of growth factors and cytokines secreted by transplanted osteoprogenitors derived from hiPSCs might explain the superior results obtained using iPSop cells encapsulated in self-assembling peptide nanofiber hydrogel rather than self-assembling peptide nanofiber hydrogel alone. In the present study, no attempts were made to examine the signalling molecules involved in the healing of the bone defects after the transplantation of iPSop cells. This aspect needs to be clarified in a future study.

Another notable result was the formation of a bony bridge between the bone defects in the calvaria. As mentioned above, the newly formed bone was usually visible around the margins of the bone defects and the intracranial periosteum. Although all of the iPSop cells transplanted into the defect might not have survived because of rejection, even with immunosuppression treatments, the combination of self-assembling peptide nanofiber hydrogel and some iPSCs surviving from rejection might have possessed better osteoconductive properties than self-assembling peptide nanofiber hydrogel alone. In this study, there are not enough data to determine whether iPSop cells survive in rat calvarial bone defects after transplantation. This aspect requires further study. In addition, further studies are needed to determine the feasibility of the auto-transplantation of osteoblasts derived from self-iPSCs.

### Conclusion

We investigated the bone-regenerative ability of iPSop cells encapsulated in a scaffold composed of self-assembling peptide nanofiber hydrogel using a rat calvarial bone defect model. iPSop cells encapsulated in self-assembling peptide nanofiber hydrogel induced good bone regeneration *in vivo*. Our results suggest that the auto-transplantation of osteoprogenitors derived from universal self-iPSCs might be an acceptable form of bone tissue engineering in the near future.
